# Ranking of a wide multidomain set of predictor variables of children obesity by machine learning variable importance techniques

**DOI:** 10.1038/s41598-021-81205-8

**Published:** 2021-01-21

**Authors:** Helena Marcos-Pasero, Gonzalo Colmenarejo, Elena Aguilar-Aguilar, Ana Ramírez de Molina, Guillermo Reglero, Viviana Loria-Kohen

**Affiliations:** 1 Nutrition and Clinical Trials Unit, GENYAL Platform IMDEA-Food Institute, CEI UAM+CSIC, 28049 Madrid, Spain; 2 Biostatistics and Bioinformatics Unit, IMDEA-Food Institute, CEI UAM+CSIC, 28049 Madrid, Spain; 3 Molecular Oncology and Nutritional Genomics of Cancer, IMDEA-Food Institute, CEI UAM+CSIC, 28049 Madrid, Spain; 4 Production and Development of Foods for Health, IMDEA-Food Institute, CEI UAM+CSIC, 28049 Madrid, Spain; 5 Department of Production and Characterization of Novel Foods. Institute of Food Science Research (CIAL), CEI UAM+CSIC, 28049 Madrid, Spain

**Keywords:** Machine learning, Obesity, Genetics, Personalized medicine

## Abstract

The increased prevalence of childhood obesity is expected to translate in the near future into a concomitant soaring of multiple cardio-metabolic diseases. Obesity has a complex, multifactorial etiology, that includes multiple and multidomain potential risk factors: genetics, dietary and physical activity habits, socio-economic environment, lifestyle, etc. In addition, all these factors are expected to exert their influence through a specific and especially convoluted way during childhood, given the fast growth along this period. Machine Learning methods are the appropriate tools to model this complexity, given their ability to cope with high-dimensional, non-linear data. Here, we have analyzed by Machine Learning a sample of 221 children (6–9 years) from Madrid, Spain. Both Random Forest and Gradient Boosting Machine models have been derived to predict the body mass index from a wide set of 190 multidomain variables (including age, sex, genetic polymorphisms, lifestyle, socio-economic, diet, exercise, and gestation ones). A consensus relative importance of the predictors has been estimated through variable importance measures, implemented robustly through an iterative process that included permutation and multiple imputation. We expect this analysis will help to shed light on the most important variables associated to childhood obesity, in order to choose better treatments for its prevention.

## Introduction

Excess body weight in children has become a major public health problem worldwide. According to the WHO European Childhood Obesity Surveillance Initiative, 1 out of 3 European children between 6 and 9 years of age were overweight or obese in 2015^1^.

Despite the unexpected plateauing of childhood obesity rates observed in developed countries^2^, Spain maintains one of the highest European rates^3^. According to the ALADINO study, the prevalence of overweight and obesity in Spanish children is 23.2% (22.4% boys, 23.9% girls) and 18.1% (20.4% boys, 15.8% girls), respectively^4^.

Childhood obesity often leads to obesity in adults, and it is considered as one of the main risk factors associated with the development of noncommunicable diseases^5^, such as type 2 diabetes mellitus; dyslipidemia; hypertension; non-alcoholic fatty liver disease; cardiovascular disease and premature mortality in adulthood. The greater the severity of obesity, the higher is the risk of cardio-metabolic diseases, mainly in children^6^.

The multifactorial etiology of obesity is well known and includes genetic susceptibility, dietary and physical activity habits, social and health factors and, especially in the case of children, a permissive and obesogenic lifestyle that begins in the mother’s womb and continues throughout childhood and adolescence^6–8^. In this respect, Machine Learning (ML) techniques are useful tools to analyze this convoluted phenomenology, as they are especially adapted to model complex, nonlinear relationships in high-dimensional data^9^. This is the case in methods like Random Forest (RF)^10^, which are based on an ensemble of decision trees built on random samples with replacement of the training set (the so-called “bagging” or bootstrap averaging of models), and with random subsets of the predictor variables used at each split in the decision trees. The prediction for new data results from averaging the prediction of all the trees in the RF. This approach allows an extensive search in the space of predictive models (even with many predictor variables), thereby increasing the accuracy of the prediction, as well as the stability against noisy variables. Overfitting is also prevented by using bootstrap subsamples with random subsets of predictor variables that decorrelate the trees. In addition, RF includes an estimation of the external prediction error, the so-called “out-of-bag” (OOB) prediction, from the training data, by averaging for each instance the predictions of the trees that were developed without that instance. More importantly, RF permits the assessment of the relative importance of the predictor variables by the calculation, for each variable, of the increased OOB error after permuting repeatedly that variable: the higher the increase in the OOB errors after permutation, the more important the variable would be^10^. This is especially interesting for explanatory purposes of the predicted endpoint.

Another robust ensemble-based ML method is Gradient Boosting Machines (GBM)^11^. In this case, the decision trees are added sequentially, where one tree is fitted to reduce the prediction error of the previous ones. Normally a stochastic version of this approach is used, using at each new added tree a random subsample (e.g. 50%) of the whole dataset, in order to decorrelate the trees and thus result in predictions with less variance. GBM are also amenable to perform variable importance calculations.

There have been some recent efforts to use ML techniques to model obesity and other body mass index (BMI)-related endpoints (for a recent review, see^12^). However, these are mostly related to adult samples, while for the case of children the work has been limited, in some cases preliminary and with restricted variable sets, in any case genetic ones^13–22^. For a recent comprehensive review of the childhood obesity field, see^23^. An interesting approach in two recent works^20,21^ is the use of electronic health record (EHR) databases to develop ML models for childhood obesity, but their objective is mainly predictive, not explanatory, no genetic variables were used, and no variable importance techniques were used to rank the predictors. Children obesity has peculiarities that make it to require specific modeling efforts, due to the huge hormonal and metabolic changes that occur in this period. Therefore, there is a lack of ML models for pediatric samples and with high-dimensional, multidomain variable sets, especially those focused on estimating the relative importance of these variables.

The use of ML to rank predictor variables by their importance has been described for e.g. non-calcified coronary burden^24^, attention-deficit and hyperactivity disorder^25^, and Crohn's disease^26^. In the case of obesity, there is one study where RF has been used to rank variables in the prediction of BMI for adolescent girls^22^, although in that case the set of variables is more restricted both in number and domains, mostly of psychological nature and with no genetic data.

Thus, for this work we set out to analyze a pediatric sample by ML and predict its BMI based on a large set of 190 variables from different domains: single nucleotide polymorphisms (SNPs), lifestyle, social, health, diet, exercise, and gestation ones. The sample was a group of schoolchildren of Madrid (Spain) enrolled in the *GENYAL study for the prevention of childhood obesity*, and here we perform a cross-sectional analysis of the baseline data*.* Using variable importance estimations, we attempted to rank the variables and identify those more strongly associated with the target, in order to better characterize the important features for children obesity. We tried both RF and GBM models, in order to assess the robustness of the estimated ranks, and derived a consensus variable importance score for all the predictors by combining the predictions of the two models. This consensus ranking will assist in developing better prevention strategies that will result in better expectations for quality of life and longevity in the future.

We have to stress at this point that we use here the term “predictor variable” in an statistical sense, where the values of one or more independent or *predictor* variables are used to obtain the value (predict) for a dependent variable (in this case BMI), through a fitted model. Given the cross-sectional nature of the data, we are actually modelling *associations* of BMI with other variables *at a given point in time*, and not forecasting *future* values of BMI given some current values of the independent variables, as it would be in a longitudinal setting.

## Results

### Exploratory analysis

Table S1 (Supplementary Material) collects the 190 predictor variables used in the analysis. They are grouped in different domains: characteristics of schoolchildren (3); genetics (1, from 11 SNPs); physical and leisure activities (24); diet, food and nutrients (80); risk factors of pregnancy and birth (39); social, health and demographic factors (43).

The average age of the 221 participants was 6.75 ± 0.73 years (52.50% were girls (n = 116) and 47.50% boys (n = 105)). According to the WHO criteria, 32.2% of the schoolchildren evaluated had excess weight (EW) (18.1% overweight and 14.1% obesity). These figures were 25.4% and 19.0% when the International Obesity Task Force (IOFT) standard or the national criteria of the Orbegozo Foundation were used, respectively.

Table 1 shows the main descriptive characteristics regarding the schoolchildren families. Regarding the nutritional status of the parents, 57.5% of the fathers and 30.4% of the mothers had EW.

**Table 1:** Main social and economic characteristics of the families.

	Father	Mother
Age (years) (x ± SD)	42.3 ± 6.7	39.5 ± 5.2
**Country of birth % (n)**		
Spain	72.8 (142)	70.6 (142)
Other (Romania, Ecuador, Colombia, Paraguay, etc.)	27.2 (53)	29.4 (59)
**Educational levels % (n)**		
No education	0.5 (1)	0.5 (1)
Primary Education	7.6 (15)	4 (8)
Secondary Education	36.5 (72)	30 (61)
Higher n	49.7 (98)	62.7 (127)
NR/UN	5.7 (11)	2.5 (5)
**Employed % (n)**		
Yes	85.3 (167)	72.6 (146)
No	14.7 (28)	27.4 (53)
**Income % (n)**		
< 12 k€	16.4 (33)	
12–18 k€	10.0 (20)	
18–24 k€	4.5 (9)	
24–30 k€	10.0 (20)	
30–36 k€	5.0 (10)	
36–42 k€	5.5 (11)	
42–48 k€	3.0 (6)	
> 48 k€	28.9 (58)	
NR/UN	16.9 (35)	

*NR/UN* no response/unknown.

The main diet, physical activity and birth characteristics of schoolchildren by sex are summarized in Table 2.

**Table 2:** Main diet, physical activity and birth and perinatal characteristics of the schoolchildren by sex.

	General		Girls		Boys		*P*
	N	x ± SD	N	x ± SD	N	x ± SD	
**Birth and perinatal characteristics**							
Birth weight (g)	160	3182.96 ± 541.93	80	3109.66 ± 466.73	80	3256.25 ± 601.99	0.087
Birth BMI (kg/m^2^)	137	12.77 ± 1.81	67	12.58 ± 1.64	70	12.95 ± 1.95	0.280
Length of breastfeeding (months)	164	9.04 ± 8.29	77	9.62 ± 8.88	87	7.74 ± 7.74	0.493
**Anthropometric data**							
Height (cm)	221	124.74 ± 6.41	105	123.75 ± 6.63	116	125.63 ± 6.10	**0.029**
Weight (kg)	221	26.60 ± 6.03	105	26.37 ± 6.07	116	26.81 ± 6.00	0.555
Fat mass (%)	218	20.59 ± 7.17	103	20.50 ± 7.60	115	20.67 ± 6.80	0.635
Muscle mass (%)	189	28.00 ± 2.98	90	27.96 ± 2.52	99	28.03 ± 3.35	0.501
WC (cm)	220	59.73 ± 7.29	104	59.73 ± 7.21	116	59.74 ± 7.40	0.621
BMI (kg/m^2^)	221	16.92 ± 2.63	105	17.04 ± 2.73	116	16.82 ± 2.55	0.448
**Blood pressure data**							
SBP (mmHg)	221	95.38 ± 9.16	105	93.82 ± 9.51	116	96.79 ± 8.62	**0.016**
DBP (mmHg)	221	63.65 ± 6.54	105	62.88 ± 6.61	116	64.35 ± 6.42	0.084
Cardiac frequency (lpm)	221	87.49 ± 11.47	105	89.96 ± 10.83	116	85.26 ± 11.61	**0.002**
**Physical and leisure activities**							
IPAC	198	1.58 ± 0.11	92	1.57 ± 0.09	106	1.60 ± 0.12	0.054
Sleeping hours	198	9.92 ± 1.09	92	9.92 ± 1.19	106	9.92 ± 1.00	0.938
TAWH (h)	224	3.74 ± 1.81	105	3.46 ± 1.62	116	4.03 ± 1.94	**0.025**
TEE (kJ/day)	198	7256.02 ± 1000.69	92	7103.49 ± 975.32	106	7388.40 ± 1008.10	**0.029**
**Intake data**							
EI (kJ/day)	201	7755.46 ± 1407.94	93	7582.32 ± 1286.16	108	7894.91 ± 1494.10	0.125
CHD (% total EI)	201	44.48 ± 5.30	93	44.43 ± 5.73	108	44.53 ± 4.93	0.900
Simple sugars (% total EI)	201	20.15 ± 4.08	93	20.17 ± 3.63	108	20.13 ± 4.45	0.539
Vegetable fibre (g)	201	18.17 ± 5.82	93	17.76 ± 5.73	108	18.52 ± 5.91	0.185
Proteins (% total EI)	201	16.55 ± 2.17	93	16.60 ± 2.16	108	16.51 ± 2.18	0.778
Fats (% total EI)	201	38.96 ± 5.02	93	38.96 ± 5.44	108	38.95 ± 4.65	0.987
SFA (% total EI)	201	13.29 ± 2.27	93	13.25 ± 2.35	108	13.33 ± 2.20	0.806
MFA (% total EI)	201	17.23 ± 3.25	93	17.20 ± 3.49	108	17.25 ± 3.04	0.927
PFA (% total EI)	201	4.84 ± 1.50	93	4.83 ± 1.57	108	4.85 ± 1.44	0.826
Cholesterol (mg/day)	201	325.99 ± 102.14	93	322.46 ± 99.66	108	329.03 ± 104.60	0.796
Calcium (mg)	201	922.02 ± 220.22	93	909.32 ± 201.94	108	932.96 ± 235.22	0.449
Iron (mg)	201	12.00 ± 3.44	93	11.98 ± 3.96	108	12.02 ± 2.95	0.218
Zinc (mg)	201	8.59 ± 1.93	93	8.40 ± 1.75	108	8.76 ± 2.06	0.198
Magnesium (mg)	201	248.73 ± 50.49	93	242.54 ± 44.68	108	254.06 ± 54.66	0.081
Phosphorus (mg)	201	1299.11 ± 237.66	93	1276.62 ± 219.51	108	1318.48 ± 251.64	0.214
Selenium (µg)	201	77.88 ± 25.42	93	76.30 ± 21.74	108	79.24 ± 28.24	0.701
Thiamine (mg)	201	1.30 ± 0.51	93	1.27 ± 0.52	108	1.32 ± 0.50	0.196
Riboflavin (mg)	201	1.84 ± 0.54	93	1.85 ± 0.62	108	1.83 ± 0.47	0.504
Folic acid (µg)	201	241.19 ± 63.24	93	237.68 ± 62.18	108	244.20 ± 64.27	0.467
Vitamin D (µg)	201	2.15 ± 1.84	93	2.21 ± 1.93	108	2.09 ± 1.76	0.665
Cereals and grains (p/d)	201	4.05 ± 1.45	93	3.95 ± 1.59	108	4.15 ± 1.32	0.069
Vegetables (p/d)	201	2.48 ± 1.05	93	2.51 ± 1.13	108	2.46 ± 0.98	0.722
Fruits (p/d)	201	1.42 ± 0.93	93	1.46 ± 0.95	108	1.39 ± 0.91	0.689
Milk and dairy products (p/d)	201	2.61 ± 0.80	93	2.59 ± 0.76	108	2.63 ± 0.84	0.850
Meats, fish and eggs (p/d)	201	2.45 ± 0.90	93	2.46 ± 0.91	108	2.44 ± 0.91	0.881
**Quality of the diet data**							
IAS	201	65.03 ± 10.84	93	65.54 ± 11.56	108	64.59 ± 10.21	0.535
Number of daily intakes	200	4.95 ± 0.66	92	4.89 ± 0.82	108	4.99 ± 0.48	0.930
KIDMED index	200	6.50 ± 1.91	93	6.51 ± 1.93	107	6.50 ± 1.90	0.863

Data expressed as mean (x) ± standard deviation (SD).

*BMI* body mass index, *WC* waist circumference, *SBP* systolic blood pressure, *DBP* diastolic blood pressure, *TEE* total energy expended, *EI* energy intake, *CHD* carbohydrate, *PFA* polyunsaturated fatty acids, *MFA* monoinsaturated fatty acid, *SFA* saturated fatty acid, *p/d* portions/day, *IPAC* individual physical activity coefficient, *TAWH* total active weekly hours, *IAS* healthy eating index

The variants distribution of the set of SNPs selected for the genetic risk score (GRS, see Methods) are presented in Table 3. These gene variants were consistent with the Hardy–Weinberg equilibrium in all the cases (*p*-values ≥ 0.05).

**Table 3:** Single Nucleotide Polymorphisms selection for the GRS design.

SNP	HWE *p* value	MAF	Genotype (%)		
rs925946	0.6997	0.2568	GG (55.91)	GT (36.82)	TT (7.27)
rs7647305	0.9316	0.1833	CC (66.97)	CT (29.41)	TT (3.62)
rs7190492	0.0704	0.3773	GG (41.82)	AG (40.91)	AA (17.27)
rs10938397	0.3367	0.4615	AA (30.77)	AG (46.15)	GG (23.08)
rs368794	0.9477	0.3416	AA (43.44)	AT (44.80)	TT (11.76)
rs1137101	0.7369	0.4295	AA (31.82)	AG (50.45)	GG (17.73)
rs17782313	0.9549	0.1705	TT (68.64)	CT (28.64)	CC (2.73)
rs2568958	0.4587	0.3688	AA (41.18)	AG (43.89)	GG (14.93)
rs10913469	0.2257	0.1886	TT (67.27)	CT (27.73)	CC (5.00)
rs7903146	0.7012	0.3402	CC (44.29)	CT (43.38)	TT (12.33)
rs6548238	0.2053	0.1977	CC (65.91)	CT (28.64)	TT (5.45)

*SNP* single nucleotide polymorphism, *HWE* Hardy–Weinberg equilibrium, *MAF* minor allele frequency.

### Random forest model and variable importance’s

**Figure 1:**
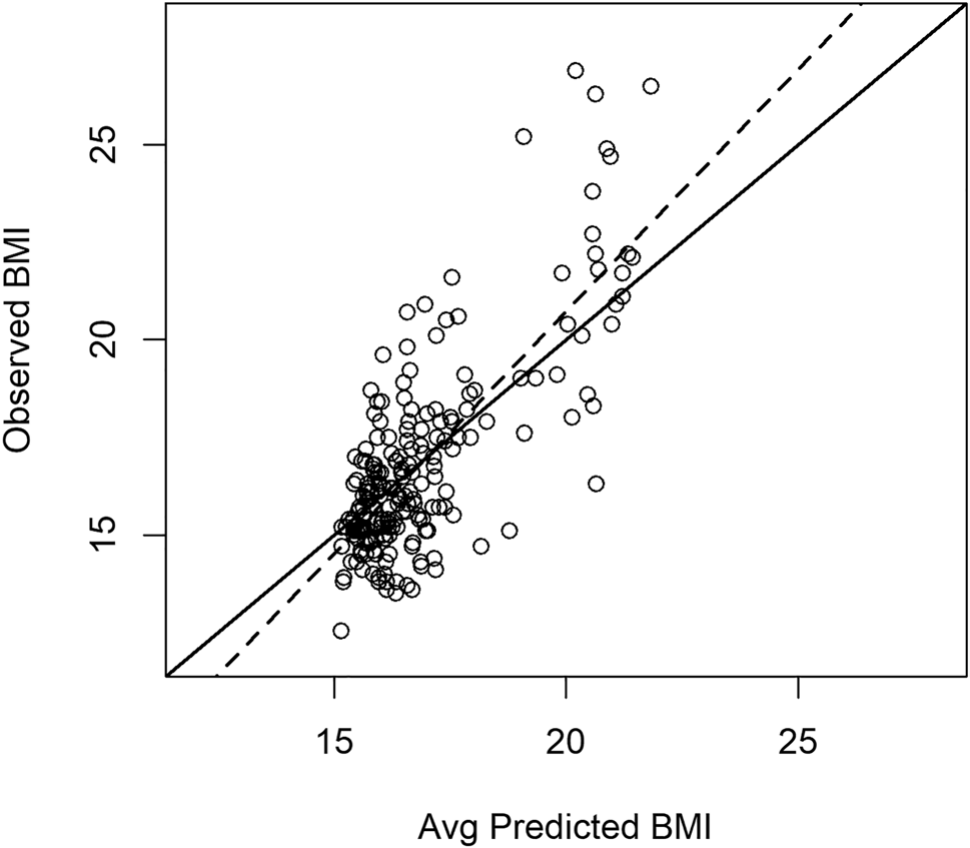
Scatter plot of the average predicted vs observed BMI for the RF models. Dashed line, best-fit line; continuous line, x = y line.

As described previously, we derived a RF model to predict the BMI in this sample. Multiple imputation was included in the calculation of the standardized importance scores *T*_*j*_ for each predictor variable *x*_*j*_ in the dataset. A total of 100 imputations were performed (see “Methods” section). On average, the RF models explain 55.07% of the variance, as estimated by the OOB pseudo-R^2^. Figure 1 shows a plot of the average predicted BMI by the RF models vs the actual BMI. We can see some degree of miscalibration in the plot, as the best-fit line (dashed line; continuous line is the x = y line) shows an intercept different from 0 (− 4.06) and a slope slightly different from 1 (1.23), so the model makes worse predictions for very high values of BMI.

**Figure 2:**
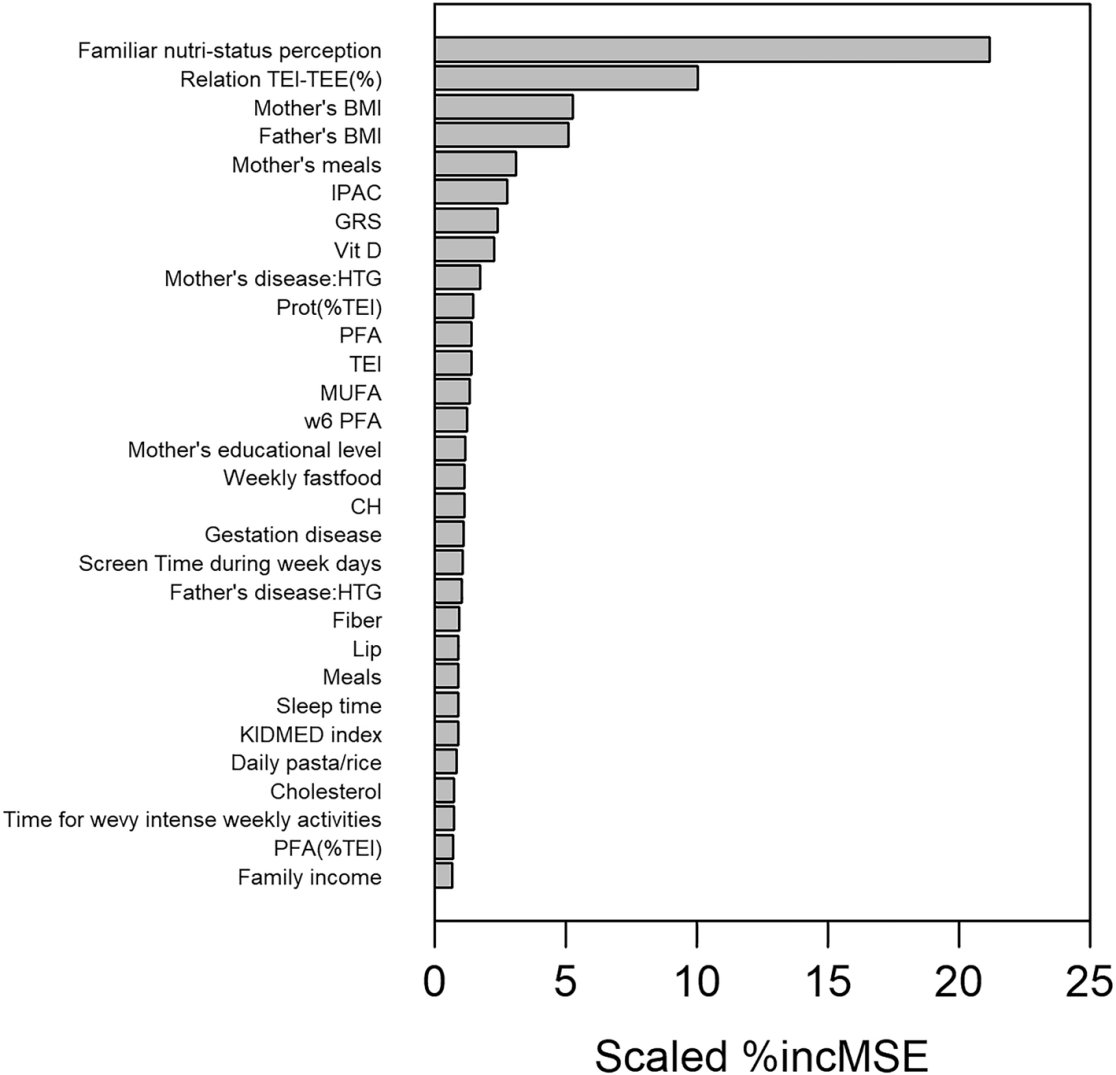
Variable importance plot of the top-30 predictor variables for the GENYAL sample, according to the RF models to predict childhood BMI.

**Figure 3:**
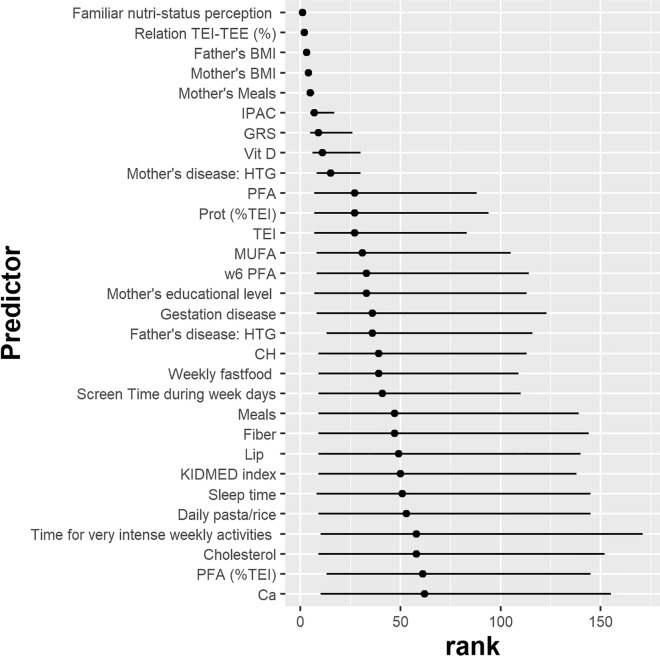
Mean average rank and 95% confidence intervals of the 30 most important predictor variables from the RF models to predict childhood BMI.

Through permutation of the OOB data, and within the imputation loop, we could obtain the scaled average variable importance of the different predictor variables. Figure 2 shows the resulting variable importance plot for the top-30 predictor variables. The use of multiple imputation allowed in addition to analyze in a robust way the variability of the rank of these variable importance’s, by estimating their mean rank and corresponding confidence intervals. Figure 3 shows the mean average rank and corresponding 95% confidence intervals of the 30 most important predictor variables**.**

The five most important variables are (in this order): *Familiar nutri-status perception* (Perception of the person completing the questionnaire about child's nutritional status) > *Relation TEI-TEE (%)* (Percentage of difference between Total Energy Intake (TEI) and Total Energy Expenditure (TEE)) > *BMI of the father* > *BMI of the mother* > *Mother’s Meals* (number of daily food servings of the mother). These variables are very well ranked, with both *Familiar nutri-status perception* and *Relation TEI-TEE (%)* having a null confidence interval in their average rank, as in all the imputations they were the first- and second-most important variables, respectively. The BMI of both parents share the same narrow confidence interval (3–4), while *Mother’s Meals* had a slightly larger confidence interval (5–7).

The next-important variables (in decreasing importance) are *IPAC* (Individual Physical Activity Coefficient) > *GRS* (genetic risk score) > *Vit D* (Vitamin D (mcg): quantity of daily vitamin D intake) > *Mother's disease: HTG* (Mother has hypertriglyceridemia by medical diagnose), with increasingly larger confidence intervals: (5–7), (5–26), (6–30) and (8–30), respectively.

The following variables show much larger confidence intervals, so that although on average they show an increasing rank, their ranking for new samples is expected to be less well defined.

### Gradient boosting machine model and relative importance’s

**Figure 4:**
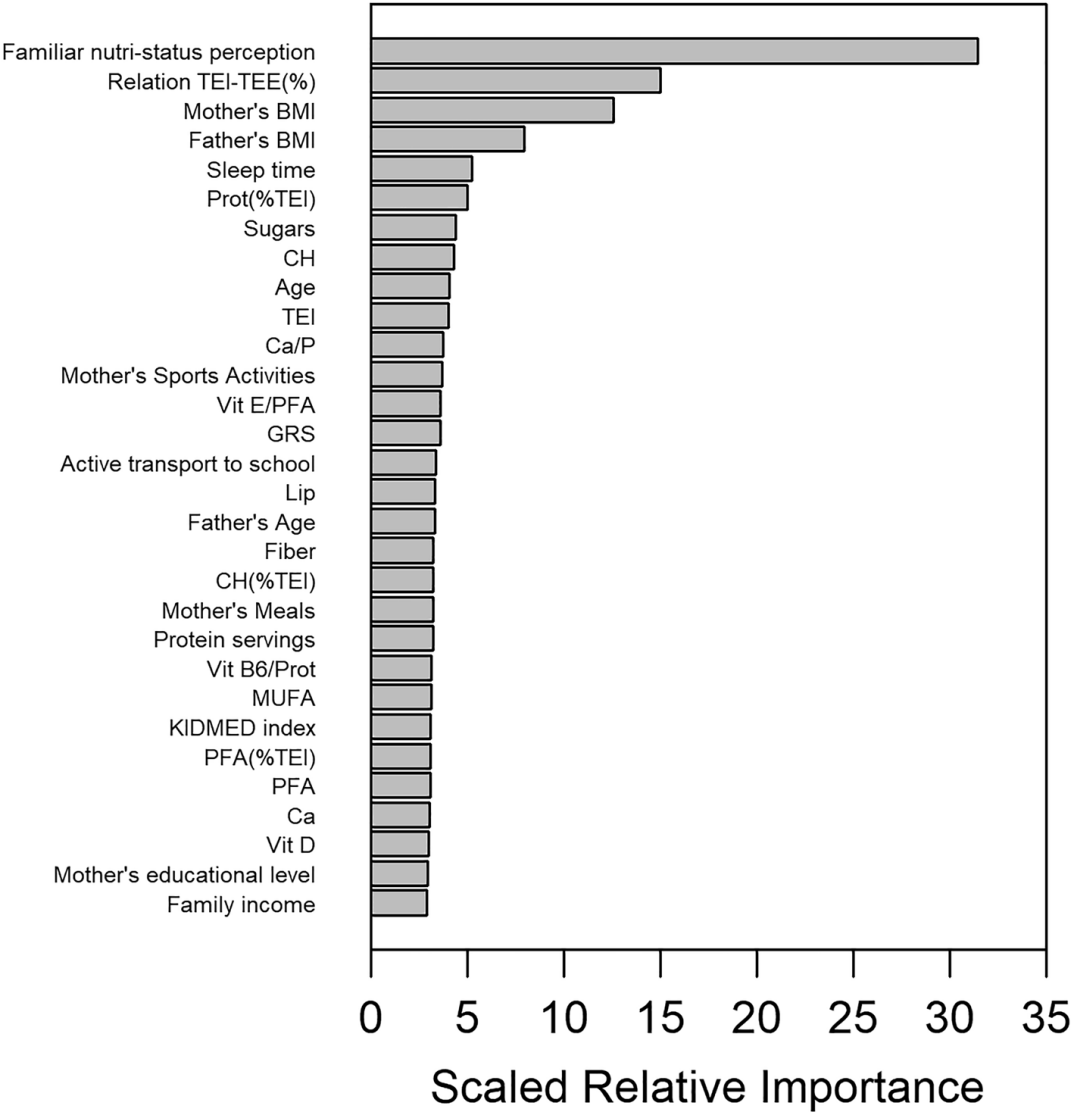
Scaled relative importance plot of the top-30 predictor variables for the GENYAL sample, according to the GBM model.

For comparison purposes, and to check the robustness of the obtained variable importance’s, an alternative method to rank the variables was used, namely scaled relative importance’s in a Gradient Boosting Machine, again implemented within an imputation loop. Figure 4 displays the corresponding scaled relative importance bar plot. We can see a rather similar picture as with RF, with 20 out of 30 top predictor variables shared between the two plots, and the four top variables (*Familiar nutri-status perception*, *Relation TEI-TEE (%), Mother’s BMI*, and *Father’s BMI*) being the same and in the same order. However, the exact ordering for the rest of the variables is not fully preserved, which is not unexpected given that the two methods use different functional forms, the metrics used to measure the importance of variables are also different, and the rankings themselves have increasing variability upon moving to less important predictor variables (e.g. Fig. 2), making unfeasible to assign an exact ranking.

### Consensus variable importance’s

**Figure 5:**
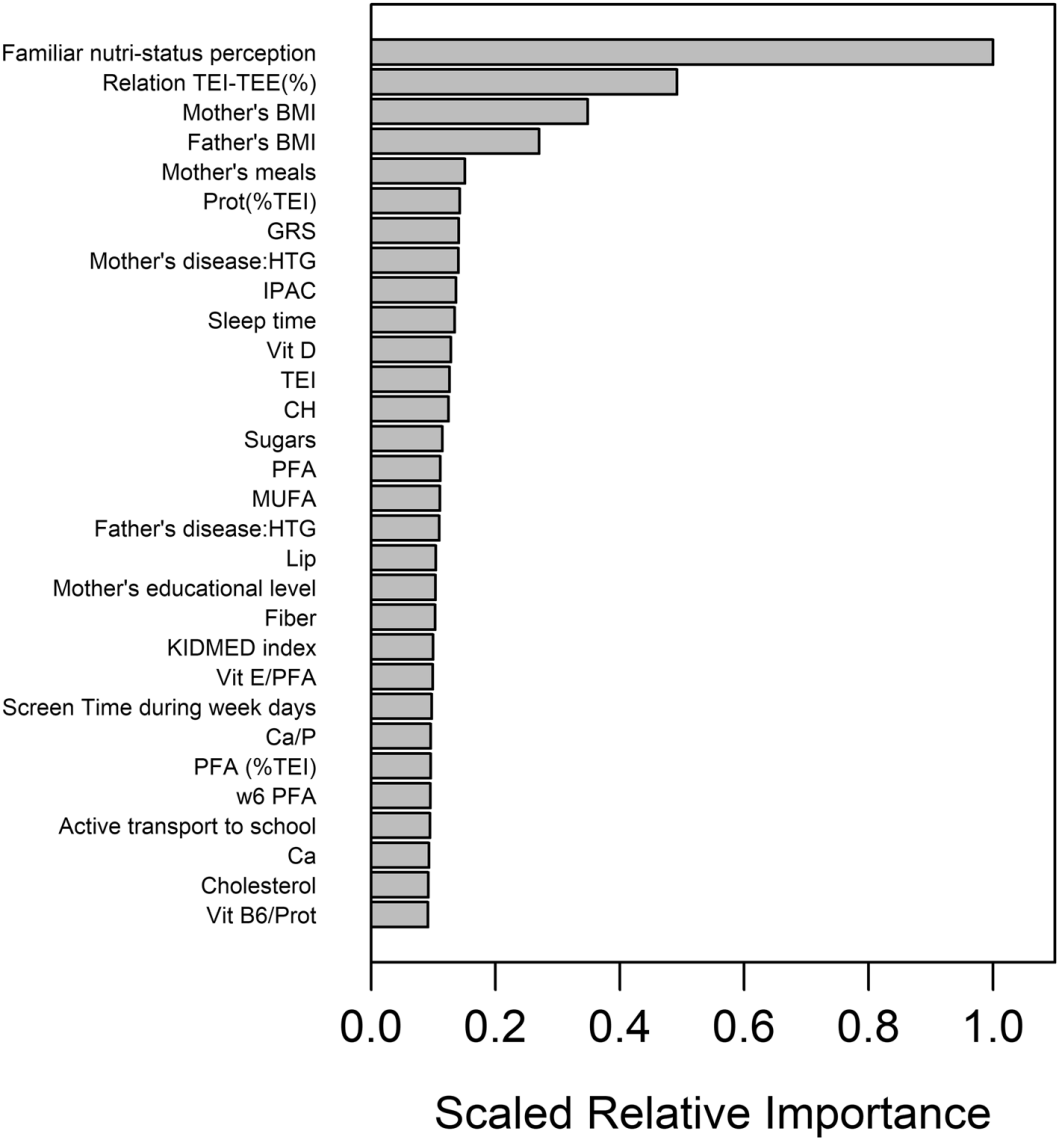
Consensus Variable Importance plot of the top-30 predictor variables for the GENYAL sample, after the RF & GBM models to predict childhood BMI.

Given that the two methods yielded reasonably similar rankings of variables, a combined variable importance was calculated for each variable by averaging the normalized variable importance matrices of the two methods. The corresponding variable importance plot is displayed in Fig. 5. Here, after the four conserved top variables (*Familiar nutri-status perception* > *Relation TEI-TEE (%)* > *Mother’s BMI* > Father’s *BMI*) the next five most important variables are, in decreasing importance, *Mother’s meals* > *Prot(%TEI)* > *GRS* > *Mother’s disease: HTG* > *IPAC.* We will focus our Discussion on this consensus score (CS) of importance’s.

## Discussion

The results of the anthropometric measurements in the current study showed that one out of four studied schoolchildren had an excess of weight. These figures, similar to those reported in the latest ALADINO national study, reflect the magnitude of the childhood obesity problem in our society^4^.

ML is a suitable approach in predictive analytics, and it has started to be used both for early preventive recommendations related to lifestyle, and to build decision-support tools for disease risk prediction^12,27^. Additionally, in view of the crucial role that prevention plays to control the high obesity prevalence, the identification of its most important risk factors could help to develop effective nutritional and educational intervention strategies. In this sense, in this study, we attempted to rank a wide set of 190 predictor variables from different domains in order to predict the BMI of children by means of ML models of the RF and GBM types.

Therefore, the novelty of the current study stems from the use of a very large number of variables from widely different domains (genetic, nutritional, exercise, social and health, lifestyle, birth and pregnancy) and their ranking by variable importance estimations. To our knowledge^23^, there is no parallel in the literature in this regard by this use of such a large multidomain set of variables for childhood obesity.

We can see that the most important variable in our CS (Fig. 5) is the *Familiar nutri-status perception*, which has not explanatory character but shows the parents awareness of the nutritional status of their children, which has anyhow a variable degree of underestimation, especially for overweight/obese children, as we (data not shown) and others have observed^28^. The next-important variable (*Relation TEI-TEE(%)*) is the questionnaire-based percentage of difference between the Total Energy Intake (TEI) and Total Energy Expenditure (TEE), which is a measure of the energy balance of the child. In this context, it is well established that obesity entails that dietary energy intake exceeds energy expenditure^29^. Nevertheless, these results should be viewed with caution, since as the literature reviewed suggests, self-reported dietary measures by questionnaires are not fully adequate to describe the energy balance^30^, and there are more accurate ways to calculate the TEE than physical activity questionnaires^31,32^. However, although non-optimal, our questionnaire-based TEI and TEE do contain valuable information about the energy input and expenditure, and thus the *Relation TEI-TEE (%)* variable results in one of the best predictors for BMI.

The following three variables of the model are *Mother’s BMI*, *Father’s BMI*, and *Mother’s Meals*. These variables would comprise genetic, diet and lifestyle aspects, indicating that children inherit to a large extent their parents’ nutritional status^33,34^. These predictors may be interesting in order to use them in predictive models for obesity even before birth, and as a matter of fact they are frequent predictor variables of simple logistic regression models for childhood obesity^23^.

The 6th variable in importance (*Prot (%TEI)*) is a measure of the percentage of protein consumption within the diet, stressing the importance of a balanced nutritional strategy to prevent obesity. *Prot (%TEI)* is followed by the genetic risk score (*GRS*), that supports the genetic component of the BMI in children. This variable aggregates several genetic single nucleotide polymorphisms well described to affect childhood obesity, and has been used previously in studies of pediatric based-populations^35,36^. GRSs have been a great success in the study on polygenic diseases, and it could be seen as a personalized risk management strategy for obesity and overweight. Similar polymorphism-based genetic scores have been described for other pathological cases like breast cancer, prostate cancer, coronary artery disease, type 1 diabetes, type 2 diabetes and Alzheimer’s disease^37,38^.

The following two variables in order of importance are mother’s hypertriglyceridemia (*Mother’s disease: HTG*) and *IPAC* score. Regarding the mother’s hypertriglyceridemia as a predicting factor for children BMI, previous studies have linked the biochemical and body composition variables between adolescents and their parents, which find significant results in BMI and total cholesterol between father and son, and hypertriglyceridemia, with inadequacies of LDL or HDL shared both by adolescents and parents^39^. In addition, the link between obesity and increased risk for hypertriglyceridemia in children has been studied^40^, and can explain the association found in this work. In turn, *IPAC* is a measure of the total physical exercise performed by the child as obtained from of the IPAC calculation, which stresses the influence of calories consumption by physical activity in the final nutritional status^41^, and nowadays, it is considered as essential focus in health promotion and obesity prevention research at early ages^42^.

As was said in the Introduction, there is a single case of ML variable importance analysis (through RF) used in the prediction task of childhood obesity^22^. The work of Rehkopf et al*.*^22^ had a longitudinal setting and the predicted endpoints were different, namely BMI percentile change after 10 years in adolescent girls, as well as transition from normal weight to overweight or obesity. The predictor variable set was more limited (41 variables) and with a more restricted set of domains: diet, physical activity, psychological, social and parent health, lacking genetic and gestational variables. In their case, psychological variables, a domain that is absent in our dataset, appeared within the most important variables; this is probably not unexpected, given that the sample was composed of adolescent girls, were this domain would be of more importance. We think that this domain would be of less importance in our 6–8 years old children.

We would like to point out some putative limitations of our study. One is the indirect nature^43,44^ of the BMI for obesity diagnosis. However, BMI is considered as a great adiposity marker and is the most practical and low-cost method, making it the most preferred one^6^. On the other hand, in pediatric samples it is frequent the use of age- and sex-specific BMI z-scores instead of raw BMI. However, our sample has a very narrow distribution of ages, with 84% of the children being 6–7 years old, and 16% 8 years old, and we did not observe significant differences between the two sexes. Therefore, we decided to use raw BMI instead, as the z-scores are quite dependent on the population they are based on.

Likewise, the use of dietary and physical activity questionnaires may lead to reporting bias and it has been criticized. To avoid or minimize such biases there is an increased need for objective measures of food intake (e.g. by use of biomarkers) and physical activity (e.g. by use of movement sensors). However, because of the high costs of such methods, questionnaires are still the most widely used instruments for determining frequency and duration of physical activity and frequency and quantity of food intake, as questionnaires are relatively cheap and efficient instruments for collecting data on a large scale in a relatively short time span^45^. Nevertheless, this information should be interpreted with caution. Another limitation was the sample size, but it is important to consider that this study is framed in an intervention study of five years and corresponds to a baseline cross-sectional analysis. Therefore, at this point this model was derived for *explanatory* purposes, in order to identify the predictors most associated to BMI. The cross-sectional nature of the present baseline dataset prevents its use from demonstration of causality, or for predictive purposes. This model rather suggests variables that would be important for childhood obesity, in order to be further tested in longitudinal settings. The new accumulated data along the study will be incorporated in order to derive models for *predictive* purposes to target appropriate preventive interventions to ameliorate effectively children obesity.

From the statistical modelling point of view, variable importance techniques can be subject to biases^46,47^. However, our use of a permutation approach avoids overestimation of categorical variables with many classes, and in preparing our dataset, we removed highly-correlated variables that could also be overestimated. In addition, the picture obtained from the GBM analysis is rather similar to the RF one, with up to 20 the 30 top variables shared variables between the two methods, and exactly the same four top variables. This gives confidence in the general conclusions above described about the influence of the different predictor variables. We must also take into account that many of these variables are correlated, so that the way that one method achieves a best fit will be different that the other given their different algorithms, while modeling basically the same physical mechanism. For instance, the important variable *IPAC* in the RF plot, is missing from the GBM plot, while in the latter *Active transport to school* instead appears. However, a large component of the physical activity of the child (measured by *IPAC*) would be going to school walking or biking, and this is measured by the *Active transport to school* variable. In the GBM plot sleep time is the fifth most important predictor, and the GRS has lower importance. In spite of that, there is a large similarity between the two descriptions of childhood obesity, taking into consideration that the dataset contains up to 190 predictor variables.

Finally, it is worth highlighting the homogeneity of the sample in terms of distribution by sex and the absence of genetic relatedness and stratification (since the Hardy–Weinberg equilibrium is met by all the SNPs). In addition, the sample shows a large representativeness with six schools from three different areas of the Community of Madrid involved, which allows to have a better knowledge of the situation throughout the Community and not from a specific school or area.

## Methods

### Study design

The GENYAL sample included 221 schoolchildren (116 girls and 105 boys) in 1st and 2nd grades (6–8 years of age) from 6 different public primary schools among the Community of Madrid (Spain). The Ministry of Education of this Community was responsible for the sampling of the schools, covering a variety of socioeconomic status of different districts, so that the selection was representative of the household income distribution in Madrid as defined by the Spanish National Statistics Institute^48^. Briefly, GENYAL is a long-term clinical trial (ClinicalTrials.gov NCT03419520) for childhood obesity prevention. The duration of the project is planned to last 5 years (2017–2021) with annual data collection, including anthropometric and nutrigenetic assessment and questionnaires about physical activity, dietary and social and health aspects. On this basis, the main objective of GENYAL study was to design and validate a predictive model that identifies those children who would benefit most from actions aimed at reducing the risk of obesity and its complications through ML algorithms. The results shown in this paper corresponding to a cross section from data collected in the first year of the study (2017).

### Ethical issues

The research was approved by the Research Ethics Committee of the IMDEA Food Foundation (PI:IM024). The study protocol follows the guidelines laid down in the Declaration of Helsinki and was performed in accordance with relevant regulations. All families signed their written informed consent to participate.

### Anthropometric measurements

Height was determined using a Leicester height rod with a millimetric accuracy (Biological Medical Technology SL, Barcelona, Spain). Body weight, fat mass percentage and muscle mass percentage were assessed using a Body Composition Monitor (BF511- OMRON HEALTHCARE Co., Ltd, Kyoto, Japan). Waist circumference were taken using a non-elastic tape (KaWe Kirchner & Wilhelm GmbH, Asperg, Germany; range 0–150 cm, 1 mm of precision). For blood pressure monitorization, an automatic digital monitor was used (OMRON M3-Intellisense) using a cuff suitable for children.

Children were measured at their schools early in the morning by trained dietitians following standard techniques and the international WHO guidelines specific for this population^49^. Measurements were taken twice in a row, considering the average as the result. BMI was calculated as weight in kg per height in squared meters; children were classified as normoweight, overweight or obese according to percentiles of the Faustino Orbegozo Foundation^50^, of the International Obesity Task Force (IOFT)^51^, and WHO growth standards^52^. The results of overweight and obesity rates were unified as a single category called excess weight (EW). Parents’ BMI was calculated from the weight and height data reported by themselves.

### SNP selection, genetic risk score and genotyping

DNA was obtained from saliva samples collected the same day of the anthropometric evaluation. Genomic DNA was extracted according to the protocol described by Stratec INVISORB Spin Tissue Mini Kit. For genotyping, the DNA samples were loaded in TaqMan OpenArray Real-Time PCR plates (Life Technologies Inc., Carlsbad, CA, USA) already configured with the specific selected SNPs with specific waves for each allele marked with a different fluorophore to determine the genotype. This process was made using the OpenArray AccuFill System (Life Technologies Inc., Carlsbad, CA, USA). Once it was charged, a PCR was made and the chips were read in the QuantStudio 12 K Flex Real-Time PCR Instrument (Life Technologies Inc., Carlsbad, CA). The results were analyzed using the TaqMan Genotyper software (Life Technologies Inc., Carlsbad, CA, USA), which assigns automatically the genotype to each sample according to the amount of detected signal for each fluorophore. Data analysis was made by TaqMan Genotyper Software v1.3 (autocaller confidence level > 90%)^53^. Call rates for all SNPs were > 96%, and genotype frequencies were in Hardy-Weingberg equilibrium (p > 0.05).

For the purpose of this study, 11 SNPs (*BDNF-AS* rs925946, *ETV5* rs7647305, *FTO* rs7190492, *GNPDA2* rs10938397, *KCTD15* rs368794, *LEPR* rs1137101 (*Q223R*), *MC4R* rs17782313, *NEGR1* rs2568958, *SEC16B* rs10913469, *TCF7L2* rs7903146 and *TMEM18* rs6548238) were selected. These SNPs were included by considering their specific relationship with childhood BMI according to previous researches, having been identified by genome-wide association studies (GWAS) and the absence of linkage disequilibrium between them. From these SNPs, a GRS was developed as the total sum of risk alleles in the 11 SNPs^53^.

### Questionnaires, data collected and predictor variables used

Different self-reported questionnaires were sent to families by email or in paper format according to the parents' preference, filled by at least one of the parents and collected by researchers. This questionnaires were based on the surveys used in previous national studies (ALADINO and ELOIN)^4,54^, KIDMED^55^, etc.

The data obtained were processed and cleaned. Finally, a total of 190 variables obtained were classified into categories according to their specific nature. **(**Table S1, supplementary material**).** These variables are described in what follows.

#### Characteristics of schoolchildren

Three variables were taken into account in this category: age, sex and school year.

#### SNP selection and GRS

The GRS, obtained from 11 SNPs variables well described as significant in childhood obesity, was used in this domain. The GRS for each child was obtained as the sum of the number of risk alleles of each of the 11 SNPs over all the SNPs, by considering that each SNP can contain 0, 1 or 2 risk alleles: e.g. if the risk allele is A, and the SNP appears as GG, GA and AA genotypes, the corresponding number of risk alleles would be 0, 1, and 2, respectively. Therefore, the GRS is defined as:

$$GRS= \sum_{i=1}^{11}{NRA}_{i}$$

Were *NRA*_*i*_ is the number of risk alleles of SNP *i*.

#### Physical and leisure activities

24 variables regarding physical activity and free time data were obtained by an ad hoc questionnaire, based on the surveys used in previous national studies (ALADINO and ELOIN), after receiving content validation by a group of dietitians and exercise science experts. A 48-h physical activity record was collected, corresponding to 24 h of a week day and a complete weekend day^56^ to obtain the Individual Physical Activity Coefficient (IPAC) and the Physical Activity Coefficient (PAC) through the coefficient defined by the WHO^49^ and by the Institute of Medicine^57^, respectively.

#### Diet, food and nutrients

80 variables were also gathered from dietary information through parent self-reported ad hoc questionnaires. These questionnaires were delivered to the parents with the corresponding filling instructions. Before processing, the responses of the questionnaires were checked by the researchers, and parents were phone called in case of unclear or omitted data. The questionnaires included were, the KIDMED validated questionnaire^55^, a 48-h food record of two non-consecutive days, a weekday and a weekend day, as recommended by the European Food Safety Authority guidelines^58^, and analyzed using the DIAL software (Alce Ingeniería, Madrid, Spain) in order to obtain information about macro and micronutrients. Finally, a questionnaire based on the surveys used in previous national studies (ALADINO and ELOIN) was used after receiving content validation by a group of Nutritionist.

#### Risk factors of pregnancy and birth

39 variables regarding the maternal and neonatal health and habits were obtained from self-reported ad hoc questionnaire completed by parents. This questionnaire was used after receiving content validation by a group of dietitians.

#### Social, health and demographic factors

43 variables were obtained from self-reported ad hoc questionnaire about the family’s status, place of birth, place of residence, etc. This questionnaire was used after receiving content validation by a group of dietitians.

### Statistical modeling

R 3.4.2 (https://www.r-project.org/) was used for all the modeling and data analysis. The sample was initially characterized by a descriptive exploratory analysis. Qualitative data were presented as percentages and absolute frequencies while quantitative data were expressed as mean ± standard deviation.

The *randomForest* package was used to develop the RF models, using as settings 500 decision trees and 5 permutations per variable for variable importance calculations. *missForest* package was used for multiple data imputation with the default settings; a total of 100 imputations were used. An iterative procedure, similar as the one described in Nonyane, et al. and Little et al.^59,60^, was applied in order to include multiple imputation in the variable importance estimation by taking into account both the between- and within-imputation variance in the importance scores. The process was as follows:

- For each imputation *m*, *m* = *1,…,M* we estimated the average importance score of variable *x*_*j*_*,* (${\widehat{\theta }}_{j}^{m}$, where *j* = *1,…,p*) as the average increase in the OOB MSE (Mean Squared Error) after OOB-permuting *x*_*j*_ for each of the B trees of the RF a total of *K* times:
  $${\widehat{\theta }}_{j}^{m}=\sum_{k=1}^{K}\sum_{b=1}^{B}({MSE}_{kbj}^{m}-{MSE}_{b}^{m})$$
  as well as the corresponding standard errors ${s}_{j}^{m}$.
- From here the average importance score across the M imputations for each variable *x*_*j*_ was obtained from:
  $${\stackrel{-}{\theta }}_{j}= \frac{1}{M}\sum_{m=1}^{M}{\widehat{\theta }}_{j}^{m}$$
- Finally, the standardized importance score for each variable *x*_*j*_ was calculated using:
  $${T}_{j}=\frac{{\widehat{\theta }}_{j}}{\sqrt{{V}_{j}}}$$
  where *V*_*j*_ is the weighted sum of the within (${\stackrel{-}{W}}_{j}$) and between (${\stackrel{-}{B}}_{j}$) imputation variances for variable *x*_*j*_:
  $${V}_{j}= {\stackrel{-}{W}}_{j}+\frac{M+1}{M}{\stackrel{-}{B}}_{j}$$
  which are defined as:
  $${\stackrel{-}{W}}_{j}= \frac{1}{M}\sum_{m=1}^{M}({{s}_{j}^{m})}^{2}$$
  $${\stackrel{-}{B}}_{j}= \frac{1}{M-1}\sum_{m=1}^{M}{({\widehat{\theta }}_{j}^{m}-{\stackrel{-}{\theta }}_{j})}^{2}$$

The multiple imputation was also used to derive (rounded to the nearest integer) mean and 95% confidence intervals for the ranks of the importance scores of the different predictor variables in the RF models.

In order to compare the results with those obtained from other methods, a Gradient Boosting Machine (GBM) relative importance plot was also obtained. The *gbm* package was used to derive the GBM models. Multiple models were derived within an imputation loop, and estimates of relative importance were pooled as described with the RF models. 100 iterations of imputation and model derivation were performed again. We used GBM models with 5000 trees, learning rate of 0.01, bag fraction of 0.5 and interaction depth of 3. The full dataset was used for training, and the best number of trees in each model was obtained through fivefold cross-validation. The relative importance of a variable *j* for a single tree *T* with *J* terminal nodes, when using regression trees in the GBM like in this case is defined as^11^

$$\hat I_j^2\left( T \right) = \mathop \sum \limits_{t = 1}^{J - 1} \hat i_t^21\left( {{v_t} = j} \right)$$

where the summation is over the nonterminal nodes *t* of the *J*-terminal node tree *T*, ${v}_{j}$ is the variable selected for splitting in that node, 1() is an indicator function that equals 1 if ${v}_{t}=j$ and 0 otherwise, and ${\widehat{i}}_{t}^{2}$ is the decrease of squared error associated to that variable. GBM is an ensemble method, were successive base learners (regression trees in our case) are fitted to minimize the residuals of the previous one; therefore, the final relative importance’s for the GBM are obtained by averaging for each variable the relative importance’s over all the trees in the model.

In order to derive a consensus variable importance’s, the two 100 imputations × 190 variable matrices of RF variable importance’s and GBM relative importance’s, were first min–max normalized (within each model) in order to make them comparable. As minimum and maximum, the minimum and maximum average variable importance (relative importance for GBM) were used, respectively. After this normalization, the two matrices were merged and averaged for each predictor variable, resulting in a normalized score for each. The top-30 scoring variables were then plotted.

## Supplementary Information

Supplementary information.
